# The association between cannabis and depression: an updated Systematic Review and Meta-analysis

**DOI:** 10.1017/S0033291724003143

**Published:** 2025-02-12

**Authors:** Victoria Churchill, Caroline Sutton Chubb, Lucy Popova, Claire A. Spears, Terri Pigott

**Affiliations:** 1 Department of Community Health and Preventive Medicine, Morehouse School of Medicine, Atlanta, GA, USA; 2 School of Public Health, Georgia State University, Atlanta, GA, USA

**Keywords:** cannabis, depression, meta-analysis, systematic review

## Abstract

**Background:**

Cannabis is one of the most commonly used drugs globally, although its legal status varies across regions. Public support for its decriminalization has increased, but gaps in our understanding of the health consequences of cannabis use remain, particularly related to its impact on mental health. This article provides an updated systematic review and meta-analysis (previous being Lev-Ran et al., 2014) looking at the relationship between cannabis and depression.

**Methods:**

Literature available before March 2023 was screened for longitudinal studies that included cannabis use and depression. Cross-sectional studies and those only looking at special populations were excluded. Studies must have also controlled for depression at baseline to allow for investigation of a temporal relationship. Extracted data included cannabis measures, depression outcomes, adjusted odds ratios, and study settings. Meta-analysis employed a random effects model with multilevel meta-regression for effect size moderators.

**Results:**

The search yielded 1,599 titles from various databases, resulting in 22 studies for meta-analysis, including 14 from Lev-Ran et al. Eleven studies were US-based, with participants mostly under 18. Meta-analysis showed a higher risk of depression among cannabis users (OR: 1.29, 95% CI: 1.13–1.46). Risk of bias assessment showed medium risk across studies with exposure measurement being a key bias area. The funnel plot and Egger’s Sandwich test did not suggest publication bias.

**Conclusions:**

This study underscores the association between cannabis use and depression but also emphasizes the need for further research, especially in understanding usage patterns, heavy use definitions, and long-term effects on depression risk amidst changing cannabis trends.

## Introduction

Cannabis is one of the most widely used drugs, with approximately 2.5% of people worldwide currently using cannabis in some form (World Drug Report 2019; World Health Organization, n.d.). As of 2019 in the United States (US), about 18% of Americans aged 12 and older reported having ever used cannabis in 2019 (Centers for Disease Control and Prevention, 2021), and in 2020, there were 2.8 million new cannabis users in the US, of which about 1 million were adolescents aged 12–17 (Substance Abuse and Mental Health Services Administration, 2020). Laws and practices surrounding cannabis have evolved significantly over the years, varying both on the state and country levels. In the US, cannabis was initially classified as a Schedule I drug under the Controlled Substances Act of 1970, indicating it had “a high potential for abuse” and “no currently accepted medical use” (Eddy, 2010). However, in the following decades, individual states enacted policies regarding the criminalization of cannabis for both personal and medical use. Recently, there has been a growing push for the decriminalization of medical and recreational cannabis (Grucza et al., 2018; Mahabir et al, 2020). Public support for cannabis decriminalization has increased sharply, and as of 2022 only 10% of adults in the US believe all cannabis should be illegal (Van Green, 2022). Nonetheless, there remain significant gaps in our understanding of the potential consequences and benefits of this drug.

One research gap is in understanding the relationship between cannabis use and mental health, specifically whether cannabis use leads to common psychological disorders such as depression. In 2014, Lev-Ran et al. published a meta-analysis investigating the development of depression among cannabis users who were not depressed at baseline (Lev-Ran et al., 2014). Their systematic review of the literature concluded in December 2012 and was not limited to any country, language, or age group. They included 14 studies that either assessed the clinical diagnosis or symptoms of major depressive disorder (MDD) or dysthymia (mild chronic depression) (Patel & Rose, 2021). They found that cannabis use was associated with higher odds of developing depression among adults (Odds Ratio [OR]: 1.17, 95% CI: 1.05, 1.30). Lev-Ran et al. also conducted meta-regressions on the age of cannabis onset (aged 18 years or younger compared to greater than 18 years) and on heavy cannabis use but found that both were not significant (*p*s > 0.05) and underpowered due to the small number of studies included. Sensitivity analyses showed that the OR for developing depression among heavy cannabis users compared to non-users was also not significant (OR: 1.34, 95% CI: 0.96–1.87). Overall, this meta-analysis indicated a positive relationship between cannabis use and the development of depression over time. No differences were found by age of onset or heaviness of cannabis use, although the latter analyses were underpowered.

Since Lev-Ran et al. (2014), there have been other systematic reviews and meta-analyses that explored the relationship between cannabis use and mental health. In 2018, Mammen and co-authors published a systematic analysis that looked at mental health outcomes among people with anxiety or other mood disorders at baseline. They found that recent cannabis use (within the prior 6 months) was associated with increased levels of mental health symptoms (Mammen et al., 2018). This offers evidence of the relationship between cannabis and poor mental health outcomes, although all participants in the Mammen et al. article had a mood disorder at baseline. Another meta-analysis published in 2019 explored cannabis use in adolescence and mental health outcomes, including depression, in young adulthood and found a pooled OR of 1.37 (95% CI: 1.16, 1.62) (Gobbi et al., 2019). This is further evidence that there is a relationship between cannabis and depression, even though the authors limited their review to only include studies with adolescent cannabis users.

Several explanations for the cannabis-depression link have been proposed. One mechanism focuses on the role of cannabis use, especially during formative periods, in changing brain structure and regulations, (Langlois et al, 2021) with some evidence from animal models (Bambico, Nguyen, Katz, & Gobbi, 2010). Another implicates shared genetic factors that might be responsible for both depression and cannabis use (Hodgson et al., 2017). Others focused on shared social or environmental factors (Degenhardt et al, 2003). Evidence supporting these mechanisms indicates that multiple pathways might be in play in explaining the relationship between cannabis and depression.

Considering the changing landscape of cannabis in the US and globally, an update to the Lev-Ran et al. meta-analysis is warranted. Besides growing social and legal acceptance of cannabis, over the years, the potency of cannabis products has been increasing (ElSohly et al, 2021; ElSohly et al., 2016). Modes of administration are also changing, with vaping cannabis becoming more popular, as well as using concentrated cannabis products (e.g., shatter, wax), all of which differ in terms of potency and delivery of the psychoactive substance (Prince & Conner, 2019). Furthermore, it is particularly important to address the issue of mental health in the US. Studies have shown that rates of depression in the US have increased in recent years, especially since the start of the COVID-19 pandemic (Niedzwiedz et al., 2021; Zheng et al., 2021). The purpose of this article was to update the Lev-Ran et al. meta-analysis by including additional studies published since 2013 and to further explore the relationship between cannabis use and depression. In addition, we conducted meta-analyses looking at the nuanced relationships between early-onset cannabis use (i.e., initiating use in adolescence) and depression, as well as heavy cannabis use and depression. We hypothesized that those who start using cannabis at a younger age would be more likely to develop depression, compared to those who initiate use as adults, which may be due to the psychosocial consequences of early cannabis use that have been demonstrated in the literature (Meier, 2021; Pacheco-Colón et al., 2019). We also expected that heavy cannabis users would be more likely to develop depression, compared to light/occasional users due to the potential for a dose–response relationship between cannabis and depression.

## Methods

This review follows the PRISMA 2020 reporting guidelines (Page et al., 2021). Although this review closely adhered to systematic review methodologies, the timing of the project did not allow for registration in the International Prospective Register of Systematic Reviews (PROSPERO).

To guide this review, we applied the PICO framework: Population (non-institutionalized youth and adults worldwide); Intervention (cannabis use); Comparison (non-cannabis users); and Outcome (depression).

### Eligibility criteria

The literature search included literature from inception through March 31, 2023. Initial eligibility criteria were established a priori, but additional criteria were added after the literature search began. Articles could originate from any country as long as they were available in English. The inclusion criteria established prior to the literature search were: (1) studies had to report on cannabis use separate from other drug use; (2) studies had to report on depression, separate from any other mental health outcomes; and (3) studies had to be longitudinal, with cannabis use measured prior to the outcome of depression or controlled for at baseline. Exclusion criteria were: (1) cross-sectional studies and (2) other systematic review and meta-analysis articles. After the preliminary search, we decided to exclude studies that only focused on special populations that were likely to have a higher risk of both cannabis use and depression, to reduce confounders. For example, LGBTQ-only studies were excluded (Dyar et al., 2020) as it has been shown that gender and sexual minority populations have higher rates of depression than cisgender and heterosexual populations (Ferlatte et al., 2020) as well as higher rates of problematic cannabis use (Dyar et al., 2021). In addition, studies that focused on war Veteran-only populations were excluded (Gunn et al., 2020) because this population also has unique experiences that may lead to differing rates of cannabis use (Davis et al., 2018) and depression (Liu et al., 2019) compared to the general population. We excluded studies that only included participants who were seeking treatment for a mental health issue at baseline (Bahorik et al., 2018).

### Search strategies

Systematic literature searches were conducted by VC and CC. The initial search started with articles that cited the Lev-Ran et al. meta-analysis. Searches were then conducted in PubMed, Ovid Medline, and Google Scholar. Gray literature was searched using ProQuest for unpublished theses and dissertations. An ancestor search of the Lev-Ran articles was conducted by reviewing all the “Cited By” articles in PubMed. The full search strategy, including keywords and limits for each database, can be found in Supplemental Materials I.

### Screening

The screening was conducted in three stages (initial screening of titles; title and abstract screening; and full-text screening) on Rayyan (Ouzzani et al., 2016) using the eligibility criteria specified above. VC conducted the original screening of potential studies published through March 31, 2022, with no specified beginning date. Both VC and CC conducted the screening of potential studies published between April 1, 2022, and March 31, 2023. Disagreements between VC and CC were discussed and TP acted as the tie-breaking vote for seven articles when agreement could not be reached. Detailed screening results, including citations of excluded articles and reasons for exclusion, can be found in Supplement II.

### Extraction

Study information was independently extracted by VC, with TP serving to assist with extracting data when clarity was needed. The coding sheet can be found in Supplement III. The final codes are detailed below.

*Cannabis Use Measure.* Cannabis use in any form was the exposure of interest. Codes specific to cannabis included: how cannabis use was measured (for example, self-reported frequency, or a validated measure such as the Composite International Diagnostic Interview [CIDI]). Specific definitions of exposure were collected. These ranged from ever use to cannabis use disorder. Most of the articles included analyses for different levels of exposure, so these were extracted as separate effect sizes. For example, Pedersen (2008) reported on participants who had used cannabis 1–10 times in the past 12 months, as well as those who used cannabis 11 or more times in the past 12 months.

Although we intended to code for the method of cannabis use (i.e., edibles, inhaled), this was not reported consistently across eligible studies. However, we were able to code for early onset/adolescent use which we defined as cannabis use prior to age 18, and heavy cannabis use. While heavy use was not universally defined, we determined that studies reporting outcomes for “cannabis dependence,” “cannabis use disorder,” “diagnosis of cannabis abuse,” “chronic cannabis use,” “persistent use” and “daily use” met the criteria for heavy use.

*Depression Measure.* The outcome was any type of clinical or self-reported depression, including major depressive disorder (MDD), major depressive episode (MDE), and general depressive symptoms. We coded for the specific measure that was used (e.g., Clinical Interview Schedule [CIS]-Revised). We also coded if the depression measure was obtained via a self-report survey, structured interview, or indeterminate. Other mental health issues, such as anxiety (Feingold, et al., 2015; Kedzior & Laeber, 2014) and schizophrenia, were not coded due to the complexity of additional codes while considering the limited resources of the project.

*Results from Individual Studies.* Adjusted odds ratios (aORs) for the depression outcomes were extracted from all studies that controlled for depression at baseline. If studies reported multiple outcomes for different exposure categories, we extracted those into separate effect sizes. For example, Feingold et al. (2015) reported aORs for those who used cannabis less than weekly, weekly to less than daily, and daily, resulting in three effects for this meta-analysis (Feingold et al., 2015). Overall, we extracted 1–5 effects from all the articles (median: 2 effects per study). When studies reported multiple aORs for a single effect, we chose the one that was adjusted for the most variables, to maintain consistency across studies. Control variables for each of the studies can be found in Table 3. We converted aOR to log-odds ratios using the R program metafor based on extant literature (Colditz et al., 1994).

*Other Coded Data.* For each study, we collected data on setting (years that the study took place, country [US vs non-US], and US state of participants), age of participants at baseline (under 18, over 18, or mix), length of follow-up period, and any adjustments made in the analyses. We also attempted to code for the percentages of gender identities in the sample, but that variable was inconsistently reported in the primary studies included in this meta-analysis.

*Risk of Bias.* The risk of bias was assessed using an adapted version of the “Tool to Assess Risk of Bias in Cohort Studies” available from Cochrane (Supplement IV) (Sterne et al., 2022). We utilized 7 out of the 8 items that applied to our systematic review, including “Can we be confident in the assessment of exposure?” We did not use the item that assessed co-interventions, since it was not appropriate for this systematic review (Supplement V). As suggested by the literature, multiple tests were used to triangulate evidence of publication bias (Vevea et al., 2019). The risk of bias was assessed by testing funnel plot asymmetry (Pustejovsky & Tipton, 2022) and conducting an Egger’s regression test adapted for dependent effect sizes (Duval & Tweedie, 2000; Egger et al., 1997).

*Meta-analytic Approach.* Rstudio version 1.2 was used to conduct all analyses (RStudio Team, 2018). We used a random effects model to synthesize the results given the likely variation across studies in population and context. The random effects models were estimated using REML. Many studies reported multiple effect sizes due to subgroup analyses (e.g., different levels of cannabis use, different ages of onset of use); thus, given the presence of dependent effect sizes, we used robust variance estimation for the analysis using the package clubSandwich (Pustejovsky & Tipton, 2022). We estimated effect size models using a hierarchical and correlated effects model assuming a correlation of 0.6 among dependent effect sizes. This allowed us to make more conservative assumptions about statistical significance. For the meta-analyses of early onset, heavy use, and country, we examined effect size moderators using multilevel meta-regression and RVE adjustment using metafor and clubSandwich. As suggested by Williams et al. (2021), these models are exploratory in nature (Williams et al, 2022).

## Results

### Search results

**Figure 1.**
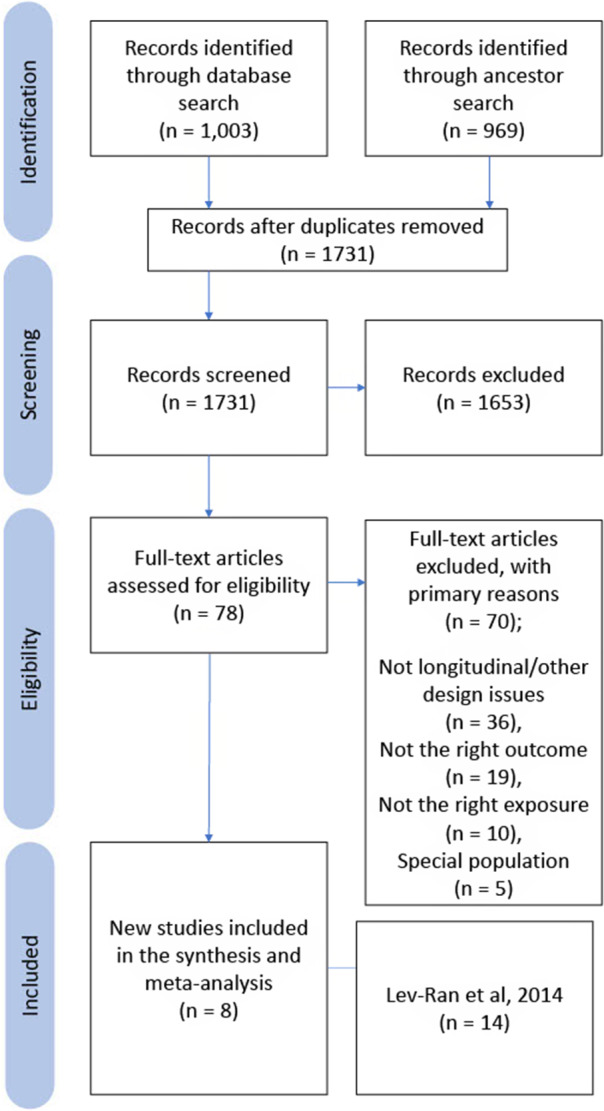
PRISMA diagram.

Overall, 1,599 titles were initially screened across PubMed (1372), MedLine (222), and ProQuest (5). From the ancestor search of the Lev-Ran articles, we screened an additional 969 articles. After duplicates were removed (n = 249) there were 2,319 articles that were title screened. We excluded 2,123 articles based on title screening, leaving 94 articles for abstract/full-text screen. The most common reasons for exclusion were review articles, included only special populations (i.e., Veterans (Gunn et al., 2020), LGBT (London-Nadeau et al., 2021)), study design (i.e., cross-sectional (Hawke et al, 2020)), or did not look at depression separately from other mental health outcomes (Spineli & Pandis, 2020). In the end, 22 studies were included in the meta-analysis, which included the 14 original articles from the Lev-Ran meta-analysis. Summaries of the included studies can be found in Tables 1 and 2. Study selection is visualized in the PRISMA diagram (Figure 1).

**Table 1.** Summary of studies included in the meta-analysis

Article	Study name	Population	Cohort demographics	Age range at enrollment; Years of enrollment and follow-up
Areseneault, 2002	Dunedin Multidisciplinary Health and Development Study	General population prospective birth cohort in New Zealand	Not reported	Birth; 1972–1999
Blanco, 2016	National Epidemiologic Survey on Alcohol and Related Conditions	Nationally representative sample of US adults	Stratified by state demographics; oversampled for Black and Hispanic individuals and for young adults (aged 18–24)	18 (no upper limit indicated); 2001–2005
Bovasso, 2001 *	Baltimore Epidemiological Catchment Area study	Probabilistic sampling of residents from Eastern Baltimore, Maryland	62% female; 63% White; oversampled participants aged 65 and older	18 (no upper limit indicated); 1994–1996
Brook, 2002 *	Children in the Community Study	Prospective longitudinal study of children in Albany and Saratoga counties in New York	50% female; 92% white	Between 1 and 10 years of age; 1983–1992
Brook, 2011 *	Unique cohort	Four-wave longitudinal study of African-American and Puerto Rican students in Grades 7–10 in New York, US	59% female; 55% African-American; 45% Puerto Rican;	Mean age: 14 years (sd = 1.3); 1990–2005
Copeland, 2022	The Great Smoky Mountains Study	Longitudinal prospective representative cohort study of children in rural North Carolina, US	48.9% female; 6.9% black; 3.7% American Indian	Age 9, 11, and 13 years; 1993–2015
Danielsson, 2016	Mental Health, Work, and Relations study	Longitudinal, population-based study of citizens residing in Stockholm, Sweden	57.9% female; Race not reported	Age 20–64; 1998–2000
Degenhardt, 2013 *	Unique cohort	Nine-wave prospective cohort study of adolescents and young adults in Victoria, Australia	53% female; Race not reported	Mean age = 14.9; 1992–2008
Feingold, 2015	National Epidemiologic Survey on Alcohol and Related Conditions	Longitudinal and nationally representative of non-institutionalized adults in the US.	55.5% female; 20.1% Black; 18.9% Hispanic; 4.5% Other	18 (no upper age indicated); 2001–2004
Fergusson, 1997 *	Christchurch Health and Development Study	Longitudinal birth cohort of children born in Christchurch, New Zealand.	49.8% female; 14.2% Maori/Pacific Islander	Birth; 1977–1995
Gage, 2015	The Avon Longitudinal Study of Parents and Children	Longitudinal prospective population-based birth cohort of infants born in Avon, United Kingdom.	58.5% female; Race not reported	Birth; 1991–1992
Georgiades, 2007 *	Ontario Child Health Study	Prospective cohort of children and adolescence living in Ontario, Canada.	48.8% female; Race not reported	Aged 14–16 years; 1983–2001
Harder, 2006*	National Longitudinal Survey of Youth of 1979	Nationally representative longitudinal survey of youth in the US.	50.6% female; Race not reported	Aged 15–22; 1979–1994
Harder, 2008 *	Unique cohort	Longitudinal cohort of first-grade children in a Mid-Atlantic metropolitan area of the US, nested within a randomized trial.	55.3% female; 72.3% black	First-grade students; 1985–2001
Hengartner, 2020	The Zurich Study	Stratified population-based longitudinal cohort of young adults from Zurich, Switzerland.	51.6% female; Race not reported	Aged 19 (males) and 20 (females); 1978–2008
Manrique-Garcia, 2012 *	Unique cohort	Nationwide survey of Swedish men examined for compulsory military training.	100% male; Race not reported	18–20 years; 1969–2001
Marmorstein, 2011 *	Minnesota Twin Family Study	Longitudinal community-based study of twins born in Minnesota.	53.8% female; 98% White	17 years old; 1972–1996
Paton, 1997*	Unique cohort	Representative longitudinal cohort of adolescents from public high schools in New York, US	Not reported	High school aged; 1971–1972
Patton, 2002 *	Unique cohort	Stratified cluster sample from schools in Victoria, Australia.	54% female; Race not reported	Aged 18–19; 1992–1998
Pedersen, 2008 *	Young in Norway Longitudinal Study	Population-based cohort of students in Norway.	54.8% female; Race not reported	Aged 12–16; 1992–2005
Schaefer, 2021	Minnesota Center for Twin and Family Research	Three longitudinal cohorts of adolescent twins in Minnesota.	Not reported	Age 11 and age 17; 1990–2006
van Laar, 2007 *	Netherlands Mental Health Survey and Incidence Study	Longitudinal stratified cohort of adults in the Netherlands.	Not reported	Aged 18–64; 1996–1999

*Notes:* ^*^indicates article was included in the Lev-Ran, 2014 meta-analysis.

**Table 2.** Measurements used in included studies

Article	Exposure (Cannabis)		Outcome (Depression)	
	Measure	Categories	Measure	Definition
Areseneault, 2002	Survey: cannabis use (no timeframe specified)	“Never” or “Once or twice” were considered non-users; “Three times or more” were considered cannabis users.	Diagnostic and Statistical Manual of Mental Disorders (DSM)-IV	Depressive disorder
Blanco, 2016	Interview: cannabis use in the prior 12 months.	No cannabis use in the past 12 months; some cannabis use in the past 12 months, but less than 1 use per month; 1 or more uses per month.	Alcohol Use Disorder and Associated Disabilities Interview Schedule (AUDADIS-IV)	Major depressive disorder (MDD)
Bovasso, 2001 *	Survey; Diagnostic Interview Schedule (DIS)	Cannabis use disorder	DSM-III	Depressive symptoms
Brook, 2002 *	Interview; frequency	Cannabis use on an 8-point scale from never to daily use.	University of Michigan Composite International Diagnostic Interview and modified DSM-IV criteria	MDD
Brook, 2011 *	Interview; frequency	Never; a few times a year or less; about once a month; several times a month; once a week or month	Checklist 90-R	Depressive symptoms
Copeland, 2022	Interview; based on DSM–5	Daily cannabis use or cannabis use disorder	DSM-V	Depressive disorder
Danielsson, 2016	Survey; frequency	Ever use; use recency: today, last week, last month, last 12 months, more than 12 months ago.	Major Depression Inventory, based on DSM-IV and International Classification of Diseases (ICD)–9 depressive symptoms	Depressive disorder
Degenhardt, 2013 *	Survey; cannabis use in the past 6 months (adolescents) or past 12 months (young adults)	Non-users; use less than weekly (occasional); weekly or more often (daily)	Clinical Interview Schedule – Revised (CIS-R)	Major depressive episode
Feingold, 2015	Survey; lifetime and past-year cannabis use	Cannabis use frequency over the past year ranged from “every day” to “once a year”; the number of joints per cannabis use day	Alcohol Use Disorder and Associated Disabilities Interview Schedule (AUDADIS-IV)	MDD
Fergusson, 1997 *	Survey; Composite International Diagnostic Interview (CIDI)	Frequency of cannabis use from age 15–16; symptoms of cannabis abuse or cannabis dependence	CIDI	MDD
Gage, 2015	Survey; frequency	Cumulative cannabis use: 0 times; 1–20 times; 21–60 times; more than 60 times	CIS-R	Unipolar depression
Georgiades, 2007 *	Survey; frequency of cannabis use in the past 6 months	Cannabis use only in adolescence; cannabis use only in adulthood; cannabis use in both adolescence and adulthood	CIDI-Short Form	MDD
Harder, 2006*	Survey; past-year cannabis use	Non-users; light users; heavy users	Center for Epidemiologic Studies Depression Scale (CES-D)	Depressive symptoms
Harder, 2008 *	Interview; CIDI	Non-users or non-problematic users; cannabis problems (dependence or nondependent abuse)	DSM-IV	Major depressive episode
Hengartner, 2020	Interview; frequency of cannabis use and age of first use	Age of first use (before or after age 15/16 years); how often cannabis was used in adolescence (less than or equal to 10 times or at least 11 times)	DSM-III-R	Major depressive episode
Manrique-Garcia, 2012 *	Survey; frequency of cannabis use	Reported level of cannabis use: never, once, 2–4 times, 5–10 times, 11–50 times, greater than 50 times.	ICD codes from inpatient hospital admissions.	Unipolar depression.
Marmorstein, 2011 *	Interview; Substance Abuse Module of the Composite International Diagnostic Interview (CIDI-SAM)	Cannabis use disorder (dependence or abuse); infrequent cannabis users (at least once per year but not more than once per month)	Structured Clinical Interview for DSM-III-Revised	MDD
Paton, 1997*	Survey; ever and past 30 days use of cannabis	Current cannabis user; ever users	Survey; six-item index (not validated)	Depressed mood
Patton, 2002 *	Survey; frequency in the past 6 and 12 months	None to less than 5 times in the previous 12 months; 5 times ever to less than weekly; 1–4 times/week; daily (men); daily (women)	Computerized Clinical Interview Schedule (CIS)-Revised	“Lower threshold than syndromes of major depression…but one where clinical intervention would still be appropriate.”
Pedersen, 2008 *	Survey; lifetime use of cannabis and past 12 months of cannabis use	No use through “more than 50 times”	Kandel and Davies’ six-item measure of depressed mood (derived from the Johns Hopkins Symptom Checklist [SCL–90])	Depressed mood
Schaefer, 2021	Computerized Substance Use Inventory (CSU); the Diagnostic Interview for Children and Adolescents (DICA)-Revised or the Substance Abuse Module of the CIDI, or a combination	No use; less than 1 time/month; 1–3 times a month; 1 to 4 times a week; every day or nearly every day; or more than once per day.	Structured Clinical Interview for the DSM (SCID)	MDD
van Laar, 2007 *	Interview; frequency of lifetime use during the period of heaviest use	Non-users (less than 5 times in the lifetime); 1–3 days per month; 1–4 days per week; almost every day.	CIDI DMS-III-R	MDD

*Notes:* ^*^Indicates article was included in the Lev-Ran, 2014 meta-analysis.

### Study characteristics

Eleven of the studies took place in the United States, three of which were national surveys. The others were based in Sweden (n = 2), Australia (n = 2); New Zealand (n = 2), The Netherlands (n = 1), United Kingdom (n = 1), Canada (n = 1), Switzerland (n = 1), and Norway (n = 1). The majority (n = 12) enrolled participants who were under 18 at the first time point. The median follow-up time was 7 years (interquartile range: 3–16 years).

### Meta-analysis

Extracted effects can be found in Table 3.

**Table 3.** Results from the systematic review

Article	Exposed group (control group)	aOR (95% CI)^a^	Control variables
Areseneault, 2002	Cannabis use by age 15 (“never” or “once or twice” users at age 15)	1.02 (0.34, 3.04)	“Childhood psychotic symptoms and use of other drugs in adolescence”
	Cannabis use by age 18 (“never” or “once or twice” users at age 18)	1.62 (1.06, 2.49)	
Blanco, 2016	Cannabis use in the past 12 months (nonusers)	0.8 (0.6, 1.1)	“Risk factors and sociodemographic characteristics”
Bovasso, 2001 *	Diagnosis of cannabis abuse at baseline^c^ (without a cannabis abuse diagnosis)	4.0 (1.23, 12.97)	“Confounding sociodemographic variables”
Brook, 2002 *	Childhood cannabis use^b^ (never users)	1.56 (1.10, 2.22)	“Sex, age, parental educational level, family income, prior episodes of MDD”
	Adolescent cannabis use^b^ (never users)	1.44 (1.08, 1.91)	
	Adult cannabis use in “early 20’s” (never users)	1.17 (0.89, 1.55)	
Brook, 2011 *	Maturing-out cannabis use (non- or low-users)	1.5 (0.9, 3.2)	Sex, ethnicity
	Late-onset cannabis use (non- or low-users)	2.3 (1.3, 4.4)	
	Chronic cannabis use^c^ (non- or low-users)	2.9 (1.7, 5.7)	
Copeland, 2022	Daily use^c^ (cumulative odds)	1.0 (0.6, 1.5)	“Sex and race/ethnicity, childhood psychiatric disorders (mood, anxiety, behavioral and noncannabis substance disorders) and other childhood adversities (low socioeconomic status, familial instability, family dysfunction, maltreatment, and peer victimization)”
	Early onset cannabis use – “before age 16”^b^ (non-users)	2.3 (0.7, 7.5)	
	Persistent cannabis use^c^ (non-users)	1.6 (0.4, 6.3)	
	Adolescent-only cannabis use^b^ (non-users)	1.0 (0.4, 2.7)	
	Adult-onset cannabis use (non-users)	2.1 (0.5, 9.0)	
Danielsson, 2016	Lifetime ever use of cannabis (never users)	0.99 (0.82, 1.17)	“Age, sex, education, place of upbringing, family tension, other illicit drug use, alcohol-related problems (AUDIT)”
Degenhardt, 2013 *	Occasional cannabis use (no use)	1.2 (0.68, 2.0)	“Sex, non-metropolitan school location, low parental education, parental divorce/separation high-risk alcohol use in the past week and other illicit substance use: any of amphetamine, cocaine or ecstasy use in the past 12 months. and clinically significant depression/anxiety in adolescence”
	Weekly cannabis use (no use)	1.7 (0.76, 3.8)	
	Daily cannabis use^c^ (no use)	2.3 (1.1, 4.5)	
	Cannabis dependence^c^ (no use)	1.9 (0.87, 4.3)	
Feingold, 2015	Less than weekly cannabis use (cannabis nonusers)	1.04 (0.65, 1.68)	“Socio-demographic variables (sex, age, educational level, household income, marital status, urbanity, and region), 12-month alcohol use disorder and other substance use disorders (non-cannabis), 12-month diagnosis of additional psychiatric disorders at baseline (e.g., personality disorders, anxiety disorders)”
	Weekly to less than almost daily use (cannabis nonusers)	0.67 (0.37, 1.22)	
	Daily or almost daily^c^ (cannabis nonusers)	0.58 (0.22, 1.51)	
Fergusson, 1997 *	Cannabis use (never users)	1.46 (1.0, 2.15)	Maternal age, family socio-economic status, gender, changes of parents, parental history of offending, childhood sexual abuse, IQ, conduct problems, self-esteem, novelty seeking, mood disorder, anxiety disorder, alcohol abuse, daily smoking, juvenile offending, parental attachment, deviant peer affiliations
Gage, 2015	Cumulative cannabis intensity (less than 20 times, 21–60 times, greater than 60 times)	1.3 (0.98, 1.72)	“Adjusting for family history of depression, gender, urban dwelling, maternal education, cigarette use, alcohol, and other illicit drug use”
Georgiades, 2007 *	Cannabis use only in adolescence^b^ (non-users)	1.48 (0.65, 3.4)	Family level: SES, single parent home, family functioning, sex; Child level: age, grade failure, medical condition, general health status, externalizing and internalizing syndrome scales
	Cannabis use only in adulthood (non-users)	2.58 (1.67, 3.99)	
	Cannabis use in adolescence and adulthood (non-users)	4.45 (2.05, 9.66)	
Harder, 2006*	Past-year cannabis users (non-users)	1.13 (0.81, 1.65)	Propensity score weighted
	Heavy cannabis user^c^ (non-users)	1.28 (0.72, 2.25)	
Harder, 2008 *	Cannabis problems- “dependence or nondependent abuse” among females^c^ (no cannabis dependent or abuse – females)	0.7 (0.2, 2.3)	Demographic, socioeconomic status, other drug use, childhood disturbances of psychological well-being, parental monitoring, and behavioral intervention status variables
	Cannabis problems- “dependence or nondependent abuse” among males^c^ (no cannabis dependent or abuse – males)	1.7 (0.8, 3.6)	
Hengartner, 2020	First use in adolescence (younger than age 15/16)^b^ (no adolescent use)	1.84 (1.26, 2.7)	“Adjusted for: assessment year (age), sex, family climate (at age 20/21), social support (at age 20/21), parental income (at age 20/21), education level (at age 20/21), drug abuse (time-variant, age 20/21–49/50), and alcohol abuse (time-variant, age 20/21–49/50)”
	First use aged 16–20 years old (no adolescent use)	1.58 (1.04, 2.38)	
	Used 1–10 times (no adolescent use)	1.59 (1.07, 2.36)	
	Used more than 10 times (no adolescent use)	1.9 (1.26, 2.87)	
Manrique-Garcia, 2012 *	Ever used cannabis (never use)	1.0 (0.7, 1.2)	“Adjusted for prior personality disorders at conscription, IQ, disturbed behavior in childhood, social adjustment, risky use of alcohol, smoking, early adulthood socioeconomic position, use of other drugs, brought up in a city”
	Used cannabis more than 50 times^c^ (never use)	0.9 (0.5, 1.6)	
Marmorstein, 2011 *	Infrequent cannabis use (no use)	0.52 (0.19, 1.4)	Adjusted for adolescent MDD, sex/gender, parental MDD, alcohol use disorders by age 17, nicotine dependence by 17, and conduct disorder
	Cannabis use disorder^c^ (no use)	2.62 (1.22, 5.65)	
Paton, 1997*	Current cannabis use, within the past 30 days (no use in the past 30 days)	1.12 (0.87, 1.45)	None
Patton, 2002 *	Five times lifetime to less than weekly (none to less than 5 times in the previous 12 months)	0.8 (0.44, 1.5)	Adjusted for parental separation, parental education, current smoking, frequency of drinking, and use of other illicit drugs
	1–4 times a week (none to less than 5 times in the previous 12 months)	1.1 (0.6, 2.0)	
	Daily (men)^c^ (none to less than 5 times in the previous 12 months)	2.2 (0.55, 2.6)	
	Daily (women)^c^ (none to less than 5 times in the previous 12 months)	5.6 (2.6, 12.0)	
Pedersen, 2008 *	1–10 times in the past 12 months (never use)	1.4 (0.8, 2.1)	“Controlled for age, gender, parental educational level, parents unemployed or receiving social welfare benefits, parental divorce, parental smoking and alcohol problems, parental support and monitoring measured at the age of 16 years, early pubertal maturation, school marks (age 16 years), conduct problems and daily smoking (ages 16 and 21 years), alcohol intoxication (age 16 years), alcohol problems (age 21 years), depression (ages 16 and 21 years), impulsivity (age 21 years), level of education (age 21 years), unemployment and income from social security, marriage/cohabitation and being a parent (age 21 years)”
	11 or more times in the past 12 months (never use)	0.9 (0.4, 2.5)	
Schaefer, 2021	Adolescent cannabis use^b^ (cumulative cannabis use)	1.16 (1.07, 1.25)	Age sex, cohort, and zygosity
van Laar, 2007 *	1–3 days a month (no cannabis use reported at baseline)	1.49 (0.82, 2.71)	Adjusted for gender, age, education, urbanicity, employment, partner status, neurotic personality, parental psychiatric history, traumatic events in childhood, lifetime alcohol use disorders or other substance use disorders, lifetime psychotic symptoms, and lifetime anxiety disorders at baseline
	1–4 days a week (no cannabis use reported at baseline)	1.79 (0.94, 3.4)	
	Almost every day^c^ (no cannabis use reported at baseline)	1.6 (0.75, 3.42)	

*Notes:* ^*^included in the initial Lev-Ran et al. article.

a adjusted for variables in column 4, unless none specified.

b sub-analysis for early onset.

c sub-analysis for heavy/problematic use.

*Mean Effect Size and Heterogeneity.* We estimated the mean effect size using a correlated and hierarchical effects model (CHE) with robust variance estimation using clubSandwich. The estimated mean log-odds of depression was 0.25 (SE = 0.06, df = 18, p < .001). This corresponds to an OR of 1.29, with a 95% CI [1.13, 1.46]. This would suggest a higher risk of developing depression among people who used cannabis at baseline.

We utilized the model to create a 95% prediction interval of 0.75 and 2.20. This is the likely range of effect size values where we would expect a randomly selected new study for the meta-analysis to fall. This is a wide interval, suggesting a high degree of uncertainty around future predictions.

### Meta-regression

To explore the heterogeneity across studies, we used the CHE model with robust variance estimation to fit a meta-regression using Country (US versus non-US), Cannabis Use (Non-heavy vs Heavy), and Onset (Late vs Early) as moderators. Table 4 presents the results of the meta-regression model with all three moderators and the associated Satterthwaite degrees of freedom. The meta-regression results test the difference between the levels of each moderator, controlling for the other moderators in the model. Only Cannabis Use was statistically significant at the p < 0.10 level, indicating that controlling for Country and Onset, heavy cannabis users had higher log odds of depression than non-heavy users. Table 4 also presents the adjusted means for each level of the three moderators, adjusted for the other moderators in the model. With the exception of US studies, the adjusted sub-group mean odds ratios were all significantly different from 1. The US studies had an average odds ratio that did not differ from 1.

**Table 4.** Moderator results from mixed-effects meta-regression model

Coefficient	Adjusted mean OR	95% CI	RVE meta-regression coefficient or β (SE)	Df
Country				
*Non-U.S.*	1.38	[1.16, 1.66]	^	
*U.S.*	1.13	[0.97, 1.32]	−0.17 (0.13)	16.73
Cannabis use				
*Non-Heavy*	1.39	[1.17, 1.66]	^	
*Heavy*	1.81	[1.37, 2.38]	0.26 (0.12)*	9.06
Onset				
*Late*	1.39	[1.16,1.66]	^	
*Early*	1.40	[1.15, 1.71]	−0.03(0.09)	7.30

*Note:* *significant at p < 0.10, ** significant at p < .01; ^ indicates reference group;

The first column reports the conditional means. The conditional means are the predicted values (OR) from a multivariable meta-regression model that simultaneously controlled for all the listed moderators (e.g., the odds ratio for U.S. studies when all other moderators are fixed at their observed mean). The standard errors (SE) were adjusted for effect size dependencies using robust variance estimation. The p values assess whether the levels of a moderator are statistically significantly different from one another, controlling for all other moderators in the model.

*Risk of Bias.* Results from this risk of bias tool can be found in Table 5. Overall, there was a medium risk of bias across all studies with a mean of 2.17 (*sd* = 0.57) on a scale from 1 (low risk of bias) to 5 (high risk of bias). All studies were assigned a “1” for “Was the selection of exposed and non-exposed cohorts drawn from the same population?” as they all selected cannabis users and non-users from the same population. The next lowest score was for whether there was adequate control for prognostic variables related to the outcome of depression (mean = 2.14), but this one also had the highest variability (*sd* = 1.03). The category that had the highest risk of bias was in the measure of the exposure, with a mean of 2.82 and the lowest variability (*sd* = 0.45). All of the studies relied on self-report from the participants to classify cannabis use. The question that assessed the measurement of the outcome had a moderate level of bias (mean = 2.34, *sd* = 0.45). Of note, only one study looked at clinical records to make a determination for depression) (Haroz et al., 2017).

**Table 5.**
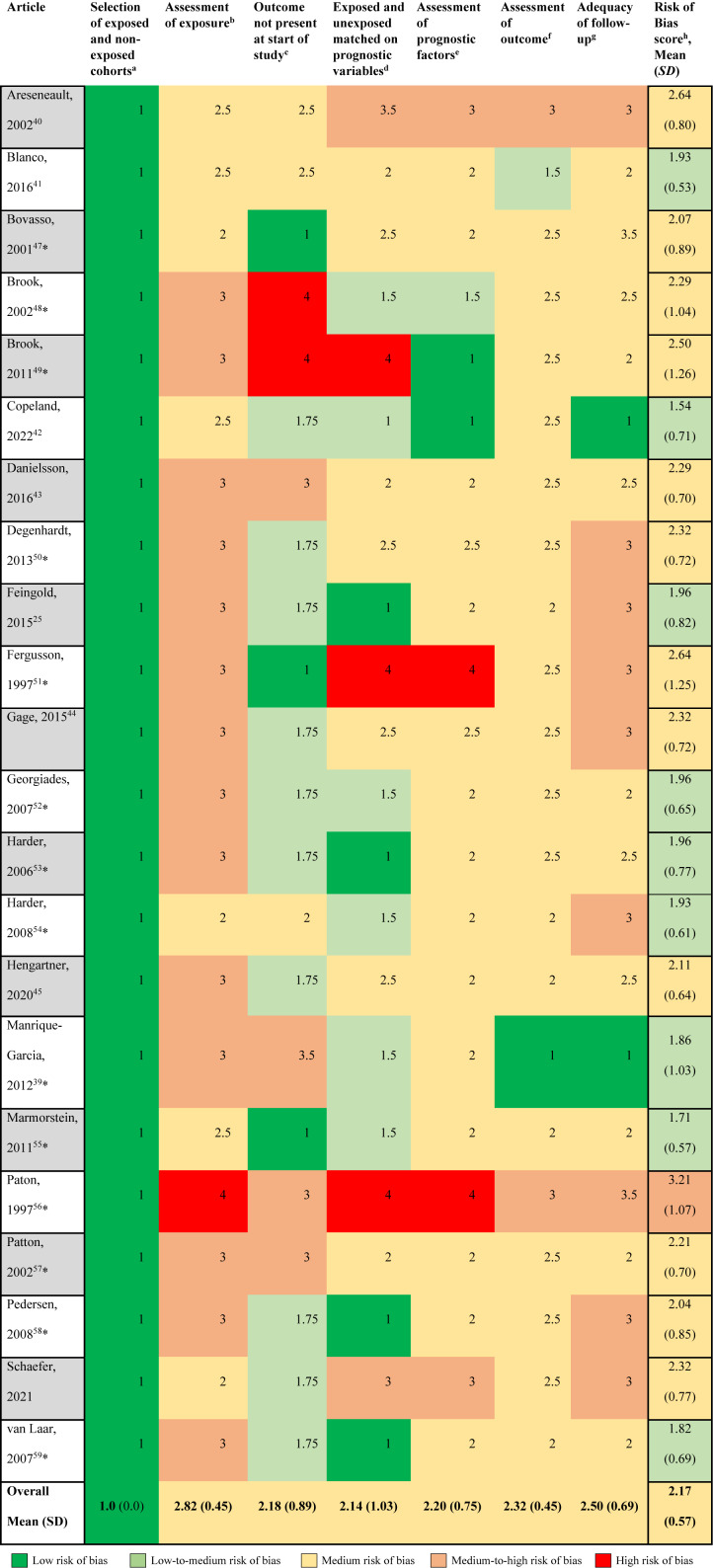
Risk of Bias assessment, average scores ranging from 1 (low risk of bias) to 5 (high risk of bias)

*Notes:* *indicates article was included in the Lev-Ran, 2014 meta-analysis. Scores were based on the following scale: 1 = “Definitely yes (low risk of bias)”; 2 = “Probably yes”; 3 = “Probably no”; 4 = “Definitely no (high risk of bias).” Half-points were given if the study was deemed to be between two levels in the risk of bias scale. *^a^*Was selection of exposed and non-exposed cohorts drawn from the same population? *^b^*Can we be confident in the assessment of the exposure? *^c^*Can we be confident that the outcome of interest was not present at start of the study? If depression at the start of the study was controlled for, we assigned a score of 1.75. *^d^*Did the study match exposed and unexposed for all variables that are associated with the outcome of interest or did the statistical analysis adjust for these prognostic variables? *^e^*Can we be confident in the assessment of the presence or abssence of prognostic factors? *^f^*Can we be confident in the assessment of outcome? *^g^*Was the follow up of cohorts adequate? *^h^*Assessed using the modified “Tool to Assess Risk of Bias in Cohort Studies” see Supplement III.

A meta-regression using items on the Risk of Bias assessment tool as predictors was conducted. We recorded each study on the Cochrane Tool metric as either high or low risk of bias, where a score of above 2.5 was considered high. We focused on two of the questions: (3) Can we be confident that the outcome of interest was not present at the start of the study?; and (4) Can we be confident in the assessment of the presence or absence of prognostic factors? These were run in separate meta-regression models as a single predictor for developing depression. Neither item was related to effect size heterogeneity.

**Figure 2.**
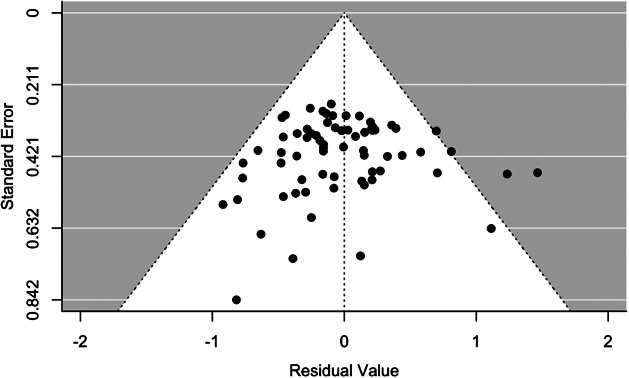
Funnel plot for publication bias.

*Selection Bias/Publication Bias.* We also produced a funnel plot (Figure 2) and conducted Egger’s sandwich regression test to check for the risk of publication bias in the presence of dependent effect sizes. The Egger’s test produced a score of 0.07, which was not significant (p = .89). This, in addition to the non-symmetric funnel plot, does not provide evidence of bias; however, it is not possible to completely rule out any risk of either selection or publication bias.

## Discussion

This systematic review and meta-analysis was designed to explore the longitudinal relationship between cannabis use and depression, building upon a prior meta-analysis by Lev-Ran et al. (2013). In updating this work, identified eight additional studies (Arseneault, 2002; Blanco et al., 2016; Copeland et al., 2022; Danielsson et al., 2016; Feingold et al., 2015; Gage et al., 2015; Hengartner et al., 2020; Schaefer et al., 2021) that we added to the original studies from the Lev-Ran et al. analysis (Bovasso, 2001; Brook et al., 2002; Brook et al., 2011; Degenhardt et al., 2013; Fergusson & Horwood, 1997; Georgiades & Boyle, 2007; Harder et al., 2008; Manrique-Garcia et al., 2012; Marmorstein & Iacono, 2011; Paton et al., 1977; Patton et al., 2002; Pedersen, 2008; van Laar et al, 2007).

Our findings indicated an odds ratio of 1.29 (95% CI: 1.13, 1.46) compared to non-users, consistent with Lev-Ran et al.’s 1.17 (95% CI: 1.05–1.30) but with a high degree of heterogeneity (95% PI: 0.75, 2.20). Similar to Lev-Ran et al., we found that studies with heavy cannabis users reported a higher odds ratio than those on non-heavy users and that the effect estimate did not significantly differ between early- and late-onset cannabis users. This lack of statistically significant difference may reflect the high heterogeneity in exposure and outcome measurements across studies, making it difficult to infer robust conclusions.

There was no significant difference in odds of depression after cannabis use when US and non-US countries were compared directly and holding the heaviness of cannabis use and onset constant. However, our interpretation of this insignificant finding remains cautious as dichotomizing US vs non-US results may not be inappropriate for the outcome of depression. For instance, a systematic review of qualitative literature exploring the definition of depression globally found that the Diagnostic and Statistical Manual of Mental Disorders (DSM) commonly used in Western countries does not adequately reflect how depression is expressed by individuals in other countries (Haroz et al., 2017; Kessler & Bromet, 2013).

### Limitations

A few key limitations should be noted. First, this review was not registered in advance with PROSPERO or a similar systematic review registry, which could have enhanced transparency and methodological rigor by establishing a priori protocols for study selection and data extraction. As such, some risk of selection bias exists, given the absence of a public pre-registration outlining the study design.

Second, we did not search all possible databases and instead relied heavily on PubMed as our primary data source. While this was in line with the previous methodology of Lev-Ran et al., it may have limited the comprehensiveness of our search and increased the risk of missing relevant studies published in other databases. Cannabis use and mental health studies, in particular, are multidisciplinary and may be published in journals indexed in databases like PsycINFO or Scopus, potentially omitting relevant literature.

Furthermore, we excluded studies focusing on specific subpopulations, such as LGBTQ+ communities, or individuals with specific psychiatric comorbidities. While this decision was made to maintain a more generalizable sample, it may have inadvertently reduced the applicability of our findings to these groups.

In summary, these limitations underscore the need for a cautious interpretation of our results, as they may not fully reflect the broader body of literature on cannabis use and depression. Future research should prioritize protocol registration, utilize a wider range of databases, and include diverse populations to provide a more comprehensive understanding of this complex relationship.

### Suggestions for future research

Throughout the process, we identified several opportunities for future research to more clearly determine the relationship between cannabis use and depression.

The definition of who is a cannabis user is not consistent across studies. For example, some studies dichotomize people into cannabis “users” and “non-users.” However, even this is not consistent across studies. The article by van Laar et al. (2007) defined a cannabis user as anyone who used it more than 5 times in their lifetime, whereas Manrique-Garcia et al. (2012) defined a cannabis user as anyone who used it at least once. Manrique-Garcia et al. had tried to categorize into more groups, but were unable to due to “the small number of cases.” Attempts to label cannabis users as “heavy,” “moderate,” and “light” were also not consistent. Furthermore, some studies looked at the past year to determine the heaviness of use, while others looked at lifetime use.

Accordingly, another limitation of the studies is that many of them were not able to consider changes in cannabis use over time. Measuring cannabis use at a single time point may not account for the waxing and waning of use across the lifecycle of an individual. While many cannabis users start in adolescence or young adulthood, there is evidence that some may discontinue the behavior as they “mature out” (Arora et al., 2021; Kosty et al., 2017). It may be beneficial to explore whether quitting cannabis as an adult reduces the risk of depression to the levels of non-users or if youth cannabis exposure is enough to elevate the risk of depression despite subsequent non-use. This could have major implications for interventions that aim to delay the onset of cannabis use. Furthermore, due to the nature of the articles included in the meta-analysis, determining an average length of follow-up is not plausible. This limits our interpretation of the role of time in the relationship between cannabis use and depression.

Similar to cannabis use, depression was inconsistently measured across studies. While “major depressive disorder” and “major depressive episode” were commonly cited, other definitions such as “depressive disorder” and “depressive symptoms” were used. Additionally, there were several diagnostic tools and scales used to measure depression across the studies; the most predominant was the DSM, with different versions (III, III-R, IV, and V) being used across studies. It is not guaranteed that an individual who met the criteria for depression on one measure would meet it on another; therefore, the heterogeneity of the outcome measure is an area of concern that future research needs to consider in order to gain a better understanding of the relationship between cannabis and depression.

Finally, we were unable to analyze differences in the relationship between cannabis use and depression across genders, racial/ethnic groups, and age groups due to insufficient reporting in the studies reviewed. This limitation highlights an important gap, as understanding how these relationships vary by gender, race/ethnicity, and age is crucial to comprehensively addressing the potential disproportionate burden of cannabis on specific demographics. Future research should prioritize collecting and reporting these demographic details to allow for more nuanced analysis and to support targeted interventions.

## Conclusions

There is evidence that cannabis use is associated with the onset of depression over time. While we updated a previous meta-analysis from 2012 and found similar results, gaps in our understanding of how cannabis affects mental health remain. The current state of the literature has not kept up with the changing landscape of cannabis use in the US and the world. Although this is partly due to the nature of longitudinal research, we recommend that researchers focus on the following: 1) consider the mode of cannabis use (e.g., edibles, concentrates); 2) find ways to better define cannabis use, specifically definitions of heavy versus light use; and 3) conduct analyses to investigate how cannabis use changes over time relates to the risk of depression.

Lastly, although our findings indicate an association, it is likely that confounding factors, which were not fully captured in our analysis, contribute to this relationship. Therefore, while our study explores a temporal, longitudinal connection, it cannot definitively establish causation.

## Supporting information

Churchill et al. supplementary material Churchill et al. supplementary material

## Supplementary material

The supplementary material for this article can be found at http://doi.org/10.1017/S0033291724003143.
