# Supplementary material for: The association between cannabis and depression: an updated Systematic Review and Meta-analysis
Source: Psychol Med. 2025 Feb 12;55:e44. doi: 10.1017/S0033291724003143 (PMC12055028; doi:10.1017/S0033291724003143)
Supplement: Churchill et al. supplementary material [file S0033291724003143sup001.docx]

# Supplemental Materials

## Supplement I: Search terms

**PubMed**

(cannabis OR marijuana AND depression OR depressive OR MDD AND longitudinal NOT retrospective NOT treatment NOT spatial NOT genetics NOT e-cigarette NOT policy NOT prenatal NOT prevalence NOT market NOT magnetic NOT parental NOT family NOT schizot* NOT schizophren* NOT receptor)

**OvidMedline**

( ( AB ( cannabis or marijuana or thc or pot or weed or thc or cbd or tetrahydrocannabinol or cannabidiol ) AND AB ( depression or depressive disorder or depressive symptoms or major depressive disorder ) AND AB ( longitudinal studies or longitudinal research or longitudinal method ) ) NOT ( self-injurious behavior or self injury or self harm ) NOT ( sexually transmitted diseases or sexually transmitted infections or sti or std ) ) NOT AB opioid

**GoogleScholar**

(cannabis OR marijuana) AND (depression or depress) AND longitudinal

**ProQuest Dissertations & Theses A&I**

ti(cannabis or marijuana) AND ti(depression or depress); limit to doctoral dissertations only

## Supplement II: Screening tool

**Original Search**

| **Citation** | **Does it mention cannabis?** | **Does it mention depression?** | **Is it a longitudinal study?** | **Does it specify cannabis use before depression?** | **Is it a special population?** | **Decision** | **Notes** |
| --- | --- | --- | --- | --- | --- | --- | --- |
| Hines LA, Freeman TP, Gage SH, et al. Association of High-Potency Cannabis Use With Mental Health and Substance Use in Adolescence. JAMA Psychiatry. 2020;77(10):1044-1051. | yes | yes | yes | yes | **yes - only cannabis users** | exclude | because it only looked at cannabis users for their analysis and did not compare cannabis users vs non users |
| Gage SH, Hickman M, Heron J, et al. Associations of Cannabis and Cigarette Use with Depression and Anxiety at Age 18: Findings from the Avon Longitudinal Study of Parents and Children. PLOS ONE. 2015;10(4):e0122896. doi:10.1371/journal.pone.0122896 | yes | yes | yes | yes | no | include |  |
| Cannabis and anxiety and depression in young adults: a large prospective study. | yes | **yes - but only in combination with anxiety** | yes | yes | no | exclude | does not separate depression from anxiety |
| Fergusson DM, Horwood LJ, Swain-Campbell NR. Cannabis dependence and psychotic symptoms in young people. Psychological Medicine. 2003;33(1):15-21. doi:10.1017/S0033291702006402 | yes | **no** | no | yes | no | exclude | "psychotic symptoms" and not depression |
| Wittchen HU, Fröhlich C, Behrendt S, et al. Cannabis use and cannabis use disorders and their relationship to mental disorders: A 10-year prospective-longitudinal community study in adolescents. Drug and Alcohol Dependence. 2007;88:S60-S70. doi:10.1016/j.drugalcdep.2006.12.013 | yes | yes | yes | **no** | no | exclude | temporal association of cannabis - depression not established |
| Fergusson DM, Boden JM. Cannabis use and later life outcomes. Addiction. 2008;103(6):969-976. doi:10.1111/j.1360-0443.2008.02221.x | yes | **no** | yes | no | no | exclude | does not look at depression |
| Blanco C, Hasin DS, Wall MM, et al. Cannabis Use and Risk of Psychiatric Disorders: Prospective Evidence From a US National Longitudinal Study. JAMA Psychiatry. 2016;73(4):388-395. doi:10.1001/jamapsychiatry.2015.3229 | yes | yes | yes | yes | no | include | it is the same population as Feingold, 2014 |
| Feingold D, Rehm J, Lev-Ran S. Cannabis use and the course and outcome of major depressive disorder: A population based longitudinal study. Psychiatry Research. 2017;251:225-234. doi:10.1016/j.psychres.2017.02.027 | yes | yes | yes | **no** | yes - people with major depressive disorder at baseline | exclude | only looked at people with MDD at baseline |
| Arseneault L. Cannabis use in adolescence and risk for adult psychosis: longitudinal prospective study. BMJ. 2002;325(7374):1212-1213. doi:10.1136/bmj.325.7374.1212 | yes | yes | yes | yes | no | Include |  |
| Hawke LD, Wilkins L, Henderson J. Early cannabis initiation: Substance use and mental health profiles of service-seeking youth. Journal of Adolescence. 2020;83:112-121. doi:10.1016/j.adolescence.2020.06.004 | yes | yes | **no** | no | yes - service seeking youth | exclude | cross-sectional and special population |
| Gunn RL, Stevens AK, Micalizzi L, Jackson KM, Borsari B, Metrik J. Longitudinal associations between negative urgency, symptoms of depression, cannabis and alcohol use in veterans. Experimental and Clinical Psychopharmacology. 2020;28(4):426-437. doi:10.1037/pha0000357 | yes | yes | yes | yes | **yes - Iraqi war veterans** | exclude | too niche of a population |
| Rasic D, Weerasinghe S, Asbridge M, Langille DB. Longitudinal associations of cannabis and illicit drug use with depression, suicidal ideation and suicidal attempts among Nova Scotia high school students. Drug and Alcohol Dependence. 2013;129(1):49-53. doi:10.1016/j.drugalcdep.2012.09.009 | **yes, but also other ilicit drugs** | yes | yes, but also concurrent | yes | no | exclude | looked at cannabis use and depression concurrently; longitudinal association was only on groups that used cannabis+other illicit drugs compared to cannabis users |
| Danielsson AK, Lundin A, Agardh E, Allebeck P, Forsell Y. Cannabis use, depression and anxiety: A 3-year prospective population-based study. Journal of Affective Disorders. 2016;193:103-108. doi:10.1016/j.jad.2015.12.045 | yes | yes | yes | yes | no | include |  |
| London-Nadeau K, Rioux C, Parent S, et al. Longitudinal associations of cannabis, depression, and anxiety in heterosexual and LGB adolescents. J Abnorm Psychol. 2021;130(4):333-345. doi:10.1037/abn0000542 | yes | yes | yes | **no** | yes | exclude | does not provide needed information on cannabis - depression |
| Hengartner MP, Angst J, Ajdacic-Gross V, Rössler W. Cannabis use during adolescence and the occurrence of depression, suicidality and anxiety disorder across adulthood: Findings from a longitudinal cohort study over 30 years. Journal of Affective Disorders. 2020;272:98-103. doi:10.1016/j.jad.2020.03.126 | yes | yes | yes | **yes** | no | include |  |
| Dyar C, Bradley H, Morgan E, Sullivan PS, Mustanski B. Reduction and Cessation of Alcohol, Cannabis, and Stimulant Use: Prospective Associations With Changes in Depressive Symptoms Across Two Cohort Studies of Sexual and Gender Minorities. J Stud Alcohol Drugs. 2020;81(6):790-797. | yes | yes | yes | yes | **yes** | exclude | looks at changes in use, which is beyond the scope of this review |
| Fergusson DM, Horwood LJ, Ridder EM. Tests of causal linkages between cannabis use and psychotic symptoms. Addiction. 2005;100(3):354-366. doi:10.1111/j.1360-0443.2005.01001.x | yes | **no** | yes | no | no | exclude | psychotic symptoms but not depression |
| Feingold D, Weiser M, Rehm J, Lev-Ran S. The association between cannabis use and mood disorders: A longitudinal study. Journal of Affective Disorders. 2015;172:211-218. doi:10.1016/j.jad.2014.10.006 | yes | yes | yes | yes | no | include |  |
| Campeny E, López-Pelayo H, Nutt D, et al. The blind men and the elephant: Systematic review of systematic reviews of cannabis use related health harms. European Neuropsychopharmacology. 2020;33:1-35. doi:10.1016/j.euroneuro.2020.02.003 | yes | yes | **no - review** | yes | no | exclude | systematic review |
| Silins E, Horwood LJ, Patton GC, et al. Young adult sequelae of adolescent cannabis use: an integrative analysis. The Lancet Psychiatry. **`**2014;1(4):286-293. doi:10.1016/S2215-0366(14)70307-4 | yes | yes | **no - review** | yes | no | exclude | meta-analysis of three cohort studies which we already included |
| Copeland WE, Hill SN, Shanahan L. Adult Psychiatric, Substance, and Functional Outcomes of Different Definitions of Early Cannabis Use. Journal of the American Academy of Child & Adolescent Psychiatry. 2022;61(4):533-543. doi:10.1016/j.jaac.2021.07.824 | yes | yes | yes | yes | no | include |  |

**Ancestor Search**

| **Parent Article** | **Citation** | **Does it mention cannabis?** | **Does it mention depression?** | **Is it a longitudinal study?** | **Does it specify cannabis use before depression?** | **Is it a special population?** | **Is it a review article?** | **Decision** | **Notes** |
| --- | --- | --- | --- | --- | --- | --- | --- | --- | --- |
| Bovasso, 2001 | Assari S, Mistry R, Caldwell CH, Zimmerman MA. Marijuana Use and Depressive Symptoms; Gender Differences in African American Adolescents. Front Psychol. 2018;9:2135. doi:10.3389/fpsyg.2018.02135 | yes | yes | yes | yes | **yes - African American** |  | exclude | path analysis rather than odds |
| Bovasso, 2001 | Bechtold J, Simpson T, White HR, Pardini D. Chronic adolescent marijuana use as a risk factor for physical and mental health problems in young adult men. Psychol Addict Behav. 2015;29(3):552-563. doi:10.1037/adb0000103 | yes | **no** | yes | no | yes - men only |  | exclude | does not provide depression outcomes |
| Bovasso, 2001 | Bornovalova MA, Verhulst B, Webber T, McGue M, Iacono WG, Hicks BM. Genetic and environmental influences on the codevelopment among borderline personality disorder traits, major depression symptoms, and substance use disorder symptoms from adolescence to young adulthood. Dev Psychopathol. 2018;30(1):49-65. doi:10.1017/S0954579417000463 | **no** |  |  |  |  |  | exclude | not cannabis |
| Bovasso, 2001 | de Graaf R, Radovanovic M, van Laar M, et al. Early cannabis use and estimated risk of later onset of depression spells: Epidemiologic evidence from the population-based World Health Organization World Mental Health Survey Initiative. Am J Epidemiol. 2010;172(2):149-159. doi:10.1093/aje/kwq096 | yes | Yes | **no - cross sectional** | yes | no |  | exclude | cross-sectional |
| Bovasso, 2001 | Dembo R, Krupa J, Wareham J, Schmeidler J, DiClemente RJ. A Multigroup, Longitudinal Study of Truant Youths, Marijuana Use, Depression, and STD-Associated Sexual Risk Behavior. J Child Adolesc Subst Abuse. 2017;26(3):192-204. doi:10.1080/1067828X.2016.1260510 | yes | **yes but not as an outcome** | yes | no | yes - truant youth |  | exlcude | not focused on cannabis - depression link |
| Bovasso, 2001 | Feingold D, Weinstein A. Cannabis and Depression. Adv Exp Med Biol. 2021;1264:67-80. doi:10.1007/978-3-030-57369-0_5 | yes | yes | **no** |  |  |  | exclude | not a study |
| Bovasso, 2001 | Gorka SM, Shankman SA, Seeley JR, Lewinsohn PM. The moderating effect of parental illicit substance use disorders on the relation between adolescent depression and subsequent illicit substance use disorders. Drug Alcohol Depend. 2013;128(1-2):1-7. doi:10.1016/j.drugalcdep.2012.07.011 | **no** | yes | yes | no | yes - youth with parents who use drugs |  | exclude | not the right population |
| Bovasso, 2001 | Kuhns L, Kroon E, Colyer-Patel K, Cousijn J. Associations between cannabis use, cannabis use disorder, and mood disorders: longitudinal, genetic, and neurocognitive evidence. Psychopharmacology (Berl). Published online November 6, 2021. doi:10.1007/s00213-021-06001-8 | yes | yes | **no - review article** |  |  |  | exclude | review article |
| Bovasso, 2001 | Lee JY, Brook JS, Kim W. Triple trajectories of alcohol use, tobacco use, and depressive symptoms as predictors of cannabis use disorders among urban adults. Psychol Addict Behav. 2018;32(4):466-474. doi:10.1037/adb00003731. Lee JY, Brook JS, Kim W. Triple trajectories of alcohol use, tobacco use, and depressive symptoms as predictors of cannabis use disorders among urban adults. Psychol Addict Behav. 2018;32(4):466-474. doi:10.1037/adb0000373 | yes | yes | yes | **no** |  |  | exclude | looks at depression - CUD |
| Bovasso, 2001 | Leventhal AM, Cho J, Stone MD, et al. Associations between anhedonia and marijuana use escalation across mid-adolescence. Addiction. 2017;112(12):2182-2190. doi:10.1111/add.13912 | yes | **no** |  |  |  |  | exclude | anhedonia not depression |
| Bovasso, 2001 | Looby A, Earleywine M. Negative consequences associated with dependence in daily cannabis users. Subst Abuse Treat Prev Policy. 2007;2:3. doi:10.1186/1747-597X-2-3 | yes | **no** |  |  |  |  | exclude | not depression |
| Bovasso, 2001 | Lucatch AM, Coles AS, Hill KP, George TP. Cannabis and Mood Disorders. Curr Addict Rep. 2018;5(3):336-345. doi:10.1007/s40429-018-0214-y | yes | yes | **no** |  |  |  | exclude | review article |
| Bovasso, 2001 | Pahl K, Brook JS, Koppel J. Trajectories of marijuana use and psychological adjustment among urban African American and Puerto Rican women. Psychol Med. 2011;41(8):1775-1783. doi:10.1017/S0033291710002345 | yes | yes | yes | **no** | yes - African American and Puerto Rican women |  | exclude | does not provide data on odds of depression after cannabis use (only average depression score) |
| Bovasso, 2001 | Rey JM, Tennant CC. Cannabis and mental health. BMJ. 2002;325(7374):1183-1184. doi:10.1136/bmj.325.7374.1183 | yes | yes | **no** | no |  |  | exclude | review article |
| Bovasso, 2001 | Smolkina M, Morley KI, Rijsdijk F, et al. Cannabis and Depression: A Twin Model Approach to Co-morbidity. Behav Genet. 2017;47(4):394-404. doi:10.1007/s10519-017-9848-0 | yes | yes | yes | **no** | yes - twins |  | exclude | does not report cannabis - depression |
| Bovasso, 2001 | Womack SR, Shaw DS, Weaver CM, Forbes EE. Bidirectional Associations Between Cannabis Use and Depressive Symptoms From Adolescence Through Early Adulthood Among At-Risk Young Men. J Stud Alcohol Drugs. 2016;77(2):287-297. doi:10.15288/jsad.2016.77.287 | yes | yes | yes | **no** | yes - at risk men |  | exclude | bidirectional and special population of men who are at risk for depression and cannabis use |
| Brook, 2002 | Brook JS, Stimmel MA, Zhang C, Brook DW. The association between earlier marijuana use and subsequent academic achievement and health problems: a longitudinal study. Am J Addict. 2008;17(2):155-160. doi:10.1080/10550490701860930 | yes | **no** |  |  |  |  | exclude | not depression |
| Brook, 2002 | Brook JS, Zhang C, Leukefeld CG, Brook DW. Marijuana use from adolescence to adulthood: developmental trajectories and their outcomes. Soc Psychiatry Psychiatr Epidemiol. 2016;51(10):1405-1415. doi:10.1007/s00127-016-1229-0 | yes | **no** |  |  |  |  | exclude | not depression |
| Brook, 2002 | Chinet L, Plancherel B, Bolognini M, et al. Substance use and depression. Comparative course in adolescents. Eur Child Adolesc Psychiatry. 2006;15(3):149-155. doi:10.1007/s00787-005-0516-1 | yes | yes | yes | **no** |  |  | exclude | looks at depression and cannabis simultaneously |
| Brook, 2002 | Chuang CWI, Chan C, Leventhal AM. Adolescent Emotional Pathology and Lifetime History of Alcohol or Drug Use With and Without Comorbid Tobacco Use. J Dual Diagn. 2016;12(1):27-35. doi:10.1080/15504263.2016.1146557 | **no** | yes | no |  |  |  | exclude | cross-sectional |
| Brook, 2002 | Fairman BJ, Anthony JC. Are early-onset cannabis smokers at an increased risk of depression spells? J Affect Disord. 2012;138(1-2):54-62. doi:10.1016/j.jad.2011.12.031 | yes | yes | **no** | yes | no |  | exclude | cross-sectional |
| Brook, 2002 | Filipponi C, Petrocchi S, Camerini AL. Bullying and Substance Use in Early Adolescence: Investigating the Longitudinal and Reciprocal Effects Over 3 Years Using the Random Intercept Cross-Lagged Panel Model. Front Psychol. 2020;11:571943. doi:10.3389/fpsyg.2020.571943 | yes | **no** |  |  |  |  | exclude | not depression |
| Brook, 2002 | Gau JM, Stice E, Rohde P, Seeley JR. Negative life events and substance use moderate cognitive behavioral adolescent depression prevention intervention. Cogn Behav Ther. 2012;41(3):241-250. doi:10.1080/16506073.2011.649781 | **No** | yes | no |  |  |  | exclude | RCT and not cannabis |
| Brook, 2002 | Green KM, Zebrak KA, Fothergill KE, Robertson JA, Ensminger ME. Childhood and adolescent risk factors for comorbid depression and substance use disorders in adulthood. Addict Behav. 2012;37(11):1240-1247. doi:10.1016/j.addbeh.2012.06.008 | yes | yes | **no** | no |  |  | exclude | not longitudinal analysis |
| Brook, 2002 | Guttmannova K, Kosterman R, White HR, et al. The association between regular marijuana use and adult mental health outcomes. Drug Alcohol Depend. 2017;179:109-116. doi:10.1016/j.drugalcdep.2017.06.016 | yes | **no** |  |  |  |  | exclude | not depression |
| Brook, 2002 | McKowen JW, Tompson MC, Brown TA, Asarnow JR. Longitudinal associations between depression and problematic substance use in the Youth Partners in Care study. J Clin Child Adolesc Psychol. 2013;42(5):669-680. doi:10.1080/15374416.2012.759226 | yes | yes | yes | **no** | Yes |  | exclude | not cannabis - depression and pts are receiving mental health tx |
| Brook, 2002 | Poudel A, Gautam S. Age of onset of substance use and psychosocial problems among individuals with substance use disorders. BMC Psychiatry. 2017;17(1):10. doi:10.1186/s12888-016-1191-0 | **no** | no | no |  |  |  | exclude | cross-sectional |
| Brook, 2002 | Schwinn TM, Schinke SP, Trent DN. Substance use among late adolescent urban youths: mental health and gender influences. Addict Behav. 2010;35(1):30-34. doi:10.1016/j.addbeh.2009.08.005 | yes | yes | **no** |  |  |  | exclude | cross-sectional |
| Brook, 2002 | Wymbs BT, McCarty CA, Mason WA, et al. Early adolescent substance use as a risk factor for developing conduct disorder and depression symptoms. J Stud Alcohol Drugs. 2014;75(2):279-289. | **yes but only with alcohol** | yes | yes | yes | no |  | exclude | doesn't look at cannabis alone |
| Degenhardt, 2013 | Botsford SL, Yang S, George TP. Cannabis and Cannabinoids in Mood and Anxiety Disorders: Impact on Illness Onset and Course, and Assessment of Therapeutic Potential. Am J Addict. 2020;29(1):9-26. doi:10.1111/ajad.12963 | yes | yes | **no** |  |  |  | Exclude | systematic review |
| Degenhardt, 2013 | Brook JS, Zhang C, Rubenstone E, Primack BA, Brook DW. Comorbid trajectories of substance use as predictors of Antisocial Personality Disorder, Major Depressive Episode, and Generalized Anxiety Disorder. Addict Behav. 2016;62:114-121. doi:10.1016/j.addbeh.2016.06.003 | **yes - but with other substances** | yes | yes | yes | no |  | exclude | does not report on cannabis alone |
| Degenhardt, 2013 | Hosseini S, Oremus M. The Effect of Age of Initiation of Cannabis Use on Psychosis, Depression, and Anxiety among Youth under 25 Years. Can J Psychiatry. 2019;64(5):304-312. doi:10.1177/0706743718809339 | yes | yes | **no** |  |  |  | exclude | systematic review |
| Degenhardt, 2013 | Keith DR, Hart CL, McNeil MP, Silver R, Goodwin RD. Frequent marijuana use, binge drinking and mental health problems among undergraduates. Am J Addict. 2015;24(6):499-506. doi:10.1111/ajad.12201 | yes | yes | **no** |  |  |  | exclude | cross-sectional |
| Degenhardt, 2013 | Renard J, Krebs MO, Le Pen G, Jay TM. Long-term consequences of adolescent cannabinoid exposure in adult psychopathology. Front Neurosci. 2014;8:361. doi:10.3389/fnins.2014.00361 | yes | **yes - but with anxiety** |  |  |  |  | exclude | does not look at depression alone |
| Degenhardt, 2013 | Rhew IC, Fleming CB, Vander Stoep A, Nicodimos S, Zheng C, McCauley E. Examination of cumulative effects of early adolescent depression on cannabis and alcohol use disorder in late adolescence in a community-based cohort. Addiction. 2017;112(11):1952-1960. doi:10.1111/add.13907 | yes | yes | yes | **no** |  |  | exclude | looks at depression - cannabis |
| Fergusson, 1997 | Brook JS, Stimmel MA, Zhang C, Brook DW. The association between earlier marijuana use and subsequent academic achievement and health problems: a longitudinal study. Am J Addict. 2008;17(2):155-160. doi:10.1080/10550490701860930 | yes | **yes - with other metal health** | yes | yes | no |  | exclude | does not separate depression from other mental health |
| Fergusson, 1997 | Goldschmidt L, Richardson GA, Larkby C, Day NL. Early marijuana initiation: The link between prenatal marijuana exposure, early childhood behavior, and negative adult roles. Neurotoxicol Teratol. 2016;58:40-45. doi:10.1016/j.ntt.2016.05.011 | **yes - prenatal exposure** | no |  |  |  |  | Exclude | prenatal exposure to cannabis |
| Fergusson, 1997 | Meier MH, Hill ML, Small PJ, Luthar SS. Associations of adolescent cannabis use with academic performance and mental health: A longitudinal study of upper middle class youth. Drug Alcohol Depend. 2015;156:207-212. doi:10.1016/j.drugalcdep.2015.09.010 | yes | **no** |  |  |  |  | exclude | "internalizing symptoms" |
| Fergusson, 1997 | Smolkina M, Morley KI, Rijsdijk F, et al. Cannabis and Depression: A Twin Model Approach to Co-morbidity. Behav Genet. 2017;47(4):394-404. doi:10.1007/s10519-017-9848-0 | yes | yes | **no** |  |  |  | exclude | genetic study |
| Georgiades, 2008 | Botsford SL, Yang S, George TP. Cannabis and Cannabinoids in Mood and Anxiety Disorders: Impact on Illness Onset and Course, and Assessment of Therapeutic Potential. Am J Addict. 2020;29(1):9-26. doi:10.1111/ajad.12963 | yes | yes | **no - review article** |  |  |  | exclude | review article |
| Georgiades, 2008 | Chadwick B, Miller ML, Hurd YL. Cannabis Use during Adolescent Development: Susceptibility to Psychiatric Illness. Front Psychiatry. 2013;4:129. doi:10.3389/fpsyt.2013.00129 | yes | yes | **no** |  |  |  | exclude | not a longitudinal study |
| Georgiades, 2008 | Esmaeelzadeh S, Moraros J, Thorpe L, Bird Y. The association between depression, anxiety and substance use among Canadian post-secondary students. Neuropsychiatr Dis Treat. 2018;14:3241-3251. doi:10.2147/NDT.S187419 | yes | yes | **no** |  |  |  | exclude | cross-sectional |
| Patton, 2002 | Fischer AS, Tapert SF, Lee Louie D, Schatzberg AF, Singh MK. Cannabis and the Developing Adolescent Brain. Curr Treat Options Psychiatry. 2020;7(2):144-161. doi:10.1007/s40501-020-00202-2 | yes | yes | **no** |  |  |  | exclude | review article |
| Patton, 2002 | Gukasyan N, Strain EC. Relationship between cannabis use frequency and major depressive disorder in adolescents: Findings from the National Survey on Drug Use and Health 2012-2017. Drug Alcohol Depend. 2020;208:107867. doi:10.1016/j.drugalcdep.2020.107867 | yes | yes | **no** |  |  |  | exclude | cross-sectional |
| Patton, 2002 | Hall W, Degenhardt L. Cannabis use and the risk of developing a psychotic disorder. World Psychiatry. 2008;7(2):68-71. doi:10.1002/j.2051-5545.2008.tb00158.x | yes | yes | **no - review article** |  |  |  | Exclude | review article |
| Patton, 2002 | Schaefer JD, Hamdi NR, Malone SM, et al. Associations between adolescent cannabis use and young-adult functioning in three longitudinal twin studies. Proc Natl Acad Sci U S A. 2021;118(14):e2013180118. doi:10.1073/pnas.2013180118 | yes | yes | yes | yes | yes - twins |  | include |  |
| Patton, 2002 | Schuler MS, Vasilenko SA, Lanza ST. Age-varying associations between substance use behaviors and depressive symptoms during adolescence and young adulthood. Drug Alcohol Depend. 2015;157:75-82. doi:10.1016/j.drugalcdep.2015.10.005 | yes | yes | **no** |  |  |  | exclude | cross-sectional |
| Patton, 2002 | Schwinn TM, Schinke SP, Trent DN. Substance use among late adolescent urban youths: mental health and gender influences. Addict Behav. 2010;35(1):30-34. doi:10.1016/j.addbeh.2009.08.005 | yes | yes | **no** |  |  |  | exclude | cross-sectional |
| Patton, 2002 | Struble CA, Ellis JD, Cairncross M, Lister JJ, Lundahl LH. Demographic, Cannabis Use, and Depressive Correlates of Cannabis Use Consequences in Regular Cannabis Users. Am J Addict. 2019;28(4):295-302. doi:10.1111/ajad.12889 | yes | **no** |  |  |  |  | exclude | not depression |
| Patton, 2002 | Wilkinson AL, Halpern CT, Herring AH, et al. Testing Longitudinal Relationships Between Binge Drinking, Marijuana Use, and Depressive Symptoms and Moderation by Sex. J Adolesc Health. 2016;59(6):681-687. doi:10.1016/j.jadohealth.2016.07.010 | **yes - but with alcohol** | yes | yes |  |  |  | exclude | looked at cannabis and alcohol predicting depression |
| Patton, 2002 | Womack SR, Shaw DS, Weaver CM, Forbes EE. Bidirectional Associations Between Cannabis Use and Depressive Symptoms From Adolescence Through Early Adulthood Among At-Risk Young Men. J Stud Alcohol Drugs. 2016;77(2):287-297. doi:10.15288/jsad.2016.77.287 | yes | yes | yes | yes | **yes - low SES/WIC receiving** |  | exclude | limited population |
| van Laar, 2007 | Chiu ML, Cheng CF, Liang WM, Lin PT, Wu TN, Chen CY. The Temporal Relationship between Selected Mental Disorders and Substance-Related Disorders: A Nationwide Population-Based Cohort Study. Psychiatry J. 2018;2018:5697103. doi:10.1155/2018/5697103 | yes | yes | yes | **no** |  |  | exclude | depression before cannabis use |
| van Laar, 2007 | Troup LJ, Andrzejewski JA, Braunwalder JT, Torrence RD. The relationship between cannabis use and measures of anxiety and depression in a sample of college campus cannabis users and non-users post state legalization in Colorado. PeerJ. 2016;4:e2782. doi:10.7717/peerj.2782 | yes | yes | **no** | no |  |  | exclude | cross-sectional |
| van Laar, 2007 | Wright NE, Scerpella D, Lisdahl KM. Marijuana Use Is Associated with Behavioral Approach and Depressive Symptoms in Adolescents and Emerging Adults. PLoS One. 2016;11(11):e0166005. doi:10.1371/journal.pone.0166005 | yes | yes | **no** |  |  |  | Exclude | cross-sectional |
| Brook, 2011 | Brook JS, Zhang C, Leukefeld CG, Brook DW. Marijuana use from adolescence to adulthood: developmental trajectories and their outcomes. Soc Psychiatry Psychiatr Epidemiol. 2016;51(10):1405-1415. doi:10.1007/s00127-016-1229-0 | yes | **no** |  |  |  |  | exclude | does not look at depression alone |
| Brook, 2011 | Caldeira KM, O’Grady KE, Vincent KB, Arria AM. Marijuana use trajectories during the post-college transition: health outcomes in young adulthood. Drug Alcohol Depend. 2012;125(3):267-275. doi:10.1016/j.drugalcdep.2012.02.022 | yes | **yes - but only provides BDI score** | yes | yes | yes - college students |  | exclude | does not provide OR information for depression, just average scores |
| Brook, 2011 | Green KM, Doherty EE, Ensminger ME. Long-term consequences of adolescent cannabis use: Examining intermediary processes. Am J Drug Alcohol Abuse. 2017;43(5):567-575. doi:10.1080/00952990.2016.1258706 | yes | **no - anxiety** |  |  |  |  | exclude | does not look at depression |

## Supplement III: Coding sheet with example

| **Article ID** | | 1 |
| --- | --- | --- |
| **Effect ID** | | 1 |
| **Article** | | Arseneault, 2002 |
| **Citation** | | Arseneault L. Cannabis use in adolescence and risk for adult psychosis: longitudinal prospective study. BMJ. 2002;325(7374):1212-1213. doi:10.1136/bmj.325.7374.1212 |
| **Study years (enrollment – final follow up)** | | 1972-1999 |
| **Setting** | | a general population birth cohort in Dunedin, New Zealand |
| **Country Code** | | 2 |
| 1 | US |  |
| 2 | Non-US |  |
| 3 | Worldwide |  |
| **State Code** | | 0 |
| 1 | National or more than 1 state |  |
| 0 | Non-US |  |
| **Ages of Participants** | | 2 |
| 1 | Adults only |  |
| 2 | Under 18 only (at baseline) |  |
| 3 | Mixed |  |
| 4 | Unclear |  |
| **Depression Definition** | | 1 |
| 1 | Major Depressive Disorder (MDD) |  |
| 2 | Major Depressive Episode (MDE) |  |
| 3 | Depressive symptoms |  |
| 4 | Depressive disorder, other |  |
| 5 | Depression, not otherwise specified |  |
| **Depression measure (open text)** | | DSM-IV |
| **Depression measure (code)** | | 1 |
| 1 | DSM and its derivatives |  |
| 2 | Symptom Checklist 90-R |  |
| 3 | Clinical Interiew Schedule (CIS-R) |  |
| 4 | CES-D |  |
| 5 | Clinical diagnosis |  |
| 6 | Symptom Distress Checklist (SCL) |  |
| 7 | Brief Symptom Inventory (BSI) |  |
| 8 | Composite International Diagnostic Interview (CIDI) |  |
| 9 | Other (specify) |  |
| **Method of Depression Assessment** | | 1 |
| 1 | Self-report |  |
| 2 | Interview |  |
| 3 | Unclear |  |
| **Cannabis Measure Code** | | 3 |
| 1 | DSM |  |
| 2 | Frequency (not a validated measure) |  |
| 3 | Dichotomized or categorizes |  |
| **Method of Cannabis Use Assessment** | | 1 |
| 1 | Self-report |  |
| 2 | Interview |  |
| 3 | Unclear |  |
| **Population (exposed group) – open text** | | Cannabis user by age 15 |
| **Heavy/Problematic Use** | | 0 |
| 1 | Heavy/Problematic |  |
| 0 | Not heavy |  |
| **Onset of Cannabis use** | | 1 |
| 1 | Early |  |
| 0 | Not early |  |
| **Follow-up period (months)** | | 468 |
| **Total (N)** | | 759 |
| **aOR** | | 1.02 |
| **95% Confidence Interval** | | 0.34 – 3.04 |
| **Adjustments/Control variables** | | childhood psychotic symptoms and se of other drugs in adolescence |
| **Risk of Bias results** | | 17.5 |

## Supplement IV: Tool to Assess Risk of Bias in Cohort Studies

**1.** **Was selection of exposed and non‐exposed cohorts drawn from the same population?**

Definitely yes Probably yes Probably no Definitely no

(low risk of bias) (high risk of bias)

Examples of low risk of bias: Exposed and unexposed drawn for same administrative data base

of patients presenting at same points of care over the same time frame

Examples of high risk of bias: exposed and unexposed presenting to different points of care or

over a different time frame

**2. Can we be confident in the assessment of exposure?**

Definitely yes Probably yes Probably no Definitely no

(low risk of bias) (high risk of bias)

Examples of low risk of bias: Secure record [e.g. surgical records, pharmacy records]; Repeated

interview or other ascertainment asking about current use/exposure

Examples of higher risk of bias: Structured interview at a single point in time; Written self

report; Individuals who are asked to retrospectively confirm their exposure status may be

subject to recall bias – less likely to recall an exposure if they have not developed an adverse

outcome, and more likely to recall an exposure (whether an exposure occurred or not) if they

have developed an adverse outcome.

Examples of high risk of bias: uncertain how exposure information obtained

**3. Can we be confident that the outcome of interest was not present at start of study**

Definitely yes Probably yes Probably no Definitely no

(low risk of bias) (high risk of bias)

**4. Did the study match exposed and unexposed for all variables that are associated with the**

**outcome of interest or did the statistical analysis adjust for these prognostic variables?**

Definitely yes Probably yes Probably no Definitely no

(low risk of bias) (high risk of bias)

Examples of low risk of bias: comprehensive matching or adjustment for all plausible prognostic

variables

Examples of higher risk of bias: matching or adjustment for most plausible prognostic variables

Examples of high risk of bias: matching or adjustment for a minority of plausible prognostic

variables, or no matching or adjustment at all. Statements of no differences between groups or

that differences were not statistically significant are not sufficient for establishing

comparability.

**5.** **Can we be confident in the assessment of the presence or absence of prognostic factors?**

Definitely yes Probably yes Probably no Definitely no

(low risk of bias) (high risk of bias)

Examples of low risk of bias: Interview of all participants; self‐completed survey from all

participants; review of charts with reproducibility demonstrated; from data base with

documentation of accuracy of abstraction of prognostic data

Examples of higher risk of bias: Chart review without demonstration of reproducibility; data

base with uncertain quality of abstraction of prognostic information

Examples of high risk of bias: Prognostic information from data base with no available

documentation of quality of abstraction of prognostic variables

**6. Can we be confident in the assessment of outcome?**

Definitely yes Probably yes Probably no Definitely no

(low risk of bias) (high risk of bias)

Examples of low risk of bias: Independent blind assessment; Record linkage; For some

outcomes (e.g. fractured hip), reference to the medical record is sufficient to satisfy the

requirement for confirmation of the fracture.

Examples of higher risk of bias: Independent assessment unblinded; self‐report; For some

outcomes (e.g. vertebral fracture where reference to x‐rays would be required) reference to

the medical record would not be adequate outcomes.

Examples of high risk of bias: uncertain (no description)

**7. Was the follow up of cohorts adequate?**

Definitely yes Probably yes Probably no Definitely no

(low risk of bias) (high risk of bias)

Examples of low risk of bias: No missing outcome data; Reasons for missing outcome data

unlikely to be related to true outcome (for survival data, censoring is unlikely to introduce bias);

Missing outcome data balanced in numbers across intervention groups, with similar reasons for

missing data across groups; For dichotomous outcome data, the proportion of missing

outcomes compared with observed event risk is not enough to have a important impact on the

intervention effect estimate; For continuous outcome data, plausible effect size (difference in

means or standardized difference in means) among missing outcomes is not large enough to

have an important impact on the observed effect size; Missing data have been imputed using

appropriate methods.

Examples of high risk of bias: Reason for missing outcome data likely to be related to true

outcome, with either imbalance in numbers or reasons for missing data across intervention

groups; For dichotomous outcome data, the proportion of missing outcomes compared with

observed event risk is enough to induce important bias in intervention effect estimate; For

continuous outcome data, plausible effect size (difference in means or standardized difference

in means) among missing outcomes is large enough to induce clinically relevant bias in the

observed effect size.

**8. Were co‐Interventions similar between groups?**

Definitely yes Probably yes Probably no Definitely no

(low risk of bias) (high risk of bias)

Examples of low risk of bias: Most or all relevant co‐interventions that might influence the

outcome of interest are documented to be similar in the exposed and unexposed.

Examples of high risk of bias: Few or no relevant co‐interventions that might influence the

outcome of interest are documented to be similar in the exposed and unexposed.

## Supplement V. Risk of Bias Results

| **Article** | **1. Was selection of exposed and non-exposed cohorts drawn from the same population?** | **2. Can we be confident in the assessment of the exposure?** | **3. Can we be confident that the outcome of interest was not present at start of the study? [if it was controlled for = 1.75)** | **4.Did the study match exposed and unexposed for all variables that are associated with the outcome of interest or did the statistical analysis adjust for these prognostic variables?** | **5. Can we be confident in the assessment of the presence or absence of prognostic factors?** | **6. Can we be confident in the assessment of outcome?** | **7. Was the follow up of cohorts adequate?** | **Mean** |
| --- | --- | --- | --- | --- | --- | --- | --- | --- |
| Arseneault, 2002 | 1 | 2.5 | 2.5 | 3.5 | 3 | 3 | 3 | **2.64** |
| Blanco, 2016 | 1 | 2.5 | 2.5 | 2 | 2 | 1.5 | 2 | **1.93** |
| Bovasso, 2001 | 1 | 2 | 1 | 2.5 | 2 | 2.5 | 3.5 | **2.07** |
| Brook, 2002 | 1 | 3 | 4 | 1.5 | 1.5 | 2.5 | 2.5 | **2.29** |
| Brook, 2011 | 1 | 3 | 4 | 4 | 1 | 2.5 | 2 | **2.50** |
| Copeland, 2022 | 1 | 2.5 | 1.75 | 1 | 1 | 2.5 | 1 | **1.54** |
| Danielsson, 2015 | 1 | 3 | 3 | 2 | 2 | 2.5 | 2.5 | **2.29** |
| Degenhardt, 2013 | 1 | 3 | 1.75 | 2.5 | 2.5 | 2.5 | 3 | **2.32** |
| Feingold, 2015 | 1 | 3 | 1.75 | 1 | 2 | 2 | 3 | **1.96** |
| Fergusson, 1997 | 1 | 3 | 1 | 4 | 4 | 2.5 | 3 | **2.64** |
| Gage, 2015 | 1 | 3 | 1.75 | 2.5 | 2.5 | 2.5 | 3 | **2.32** |
| Georglades, 2007 | 1 | 3 | 1.75 | 1.5 | 2 | 2.5 | 2 | **1.96** |
| Harder, 2006 | 1 | 3 | 1.75 | 1 | 2 | 2.5 | 2.5 | **1.96** |
| Harder, 2008 | 1 | 2 | 2 | 1.5 | 2 | 2 | 3 | **1.93** |
| Hengartner, 2020 | 1 | 3 | 1.75 | 2.5 | 2 | 2 | 2.5 | **2.11** |
| Manrique-Garcia, 2012 | 1 | 3 | 3.5 | 1.5 | 2 | 1 | 1 | **1.86** |
| Marmorstein, 2011 | 1 | 2.5 | 1 | 1.5 | 2 | 2 | 2 | **1.71** |
| Paton, 1977 | 1 | 4 | 3 | 4 | 4 | 3 | 3.5 | **3.21** |
| Patton, 2002 | 1 | 3 | 3 | 2 | 2 | 2.5 | 2 | **2.21** |
| Pedersen, 2008 | 1 | 3 | 1.75 | 1 | 2 | 2.5 | 3 | **2.04** |
| Schaefer, 2021 | 1 | 2 | 1.75 | 3 | 3 | 2.5 | 3 | **2.32** |
| van Laar, 2007 | 1 | 3 | 1.75 | 1 | 2 | 2 | 2 | **1.82** |
| **Overall** | **1** | **2.82** | **2.18** | **2.14** | **2.20** | **2.32** | **2.50** | **2.17** |
